# Pulsed Multifrequency Excitation and Spectrogram Eddy Current Testing (PMFES-ECT) for Nondestructive Evaluation of Conducting Materials

**DOI:** 10.3390/ma14185311

**Published:** 2021-09-15

**Authors:** Jacek Michał Grochowalski, Tomasz Chady

**Affiliations:** Faculty of Electrical Engineering, West Pomeranian University of Technology, 70-313 Szczecin, Poland

**Keywords:** multifrequency excitation and spectrogram eddy current testing, pulsed eddy current testing, nondestructive testing

## Abstract

This paper presents a new method for nondestructive testing—a pulsed multifrequency excitation and spectrogram eddy current testing (PMFES-ECT), which is an extension of the multifrequency excitation and spectrogram eddy current testing. The new method uses excitation in the form of pulses repeated at a specified time, containing several periods of a waveform consisting of the sum of sinusoids with a selected frequency, amplitude and phase. This solution allows the maintenance of the advantages of multifrequency excitation and, at the same time, generates high energy pulses similar to those used in pulse eddy current testing (PECT). The effectiveness of the new method was confirmed by numerical simulations and the measurement of thin Inconel plates, consisting of notches manufactured by the electric-discharge method.

## 1. Introduction

NDT (nondestructive testing) refers to the many techniques for assessing components or systems’ technical conditions and properties without causing damage. Standard NDT methods are X-ray testing, ultrasonic testing, magnetic flux leakage testing and eddy current testing. Hidden defects, cracks and stresses are inherent in the production and use of structural elements, and therefore the detection and assessment of this damage is essential.

Eddy current testing (ETC) has been used as a nondestructive testing method in the aerospace [1], petrochemical [2] and shipbuilding industries. It is used for surface inspection [3], quality inspection [4] and thickness measurements [5], among other things. Reasonable sensitivity, high speed of measurement and affordability are its main advantages.

The earliest version of ETC is the single frequency method using coils as the excitation element due to the simplicity of implementation [6]. The single-frequency eddy current technique with a phase analysis has limited potential for the inspection of surface defects in inhomogeneous ferromagnetic materials, as signals from cracks are hidden in noise caused by the lift-off of the probe or local changes to the tested specimen’s permeability [7]. In the eddy current tests, the impedance plane plots show that the phase angles between the impedance planes change with the frequency.

The phase angles between lift-off, cracks and permeability variations may be at one set of values at one frequency and another set of values at a different frequency. Two excitation frequencies used simultaneously in the test coil allow the reduction of the unwanted influence of external structures (i.e., support elements). Dual-frequency EC testing is currently standard, but up to eight frequencies are processed and analyzed simultaneously in many systems. Two testing frequencies were applied to ensure good phase discrimination. One frequency is likely to be about ten times the other. The phase and impedance sensitivity for each frequency is then adjusted independently on the vector display. A dual-frequency ECT system for detecting cracks in welds was proposed in [7]. Changes in the amplitude ratio and phase difference were utilized to enhance defect detection. Dual-frequency systems [8] with the use of data fusion were discussed in [8]. The changes in impedance for two different frequencies were analyzed and the resulting images were combined. Dual-frequency systems create the possibility of detecting surface and hidden defects. In patent [9], an extended system using four different test frequencies sequentially applied to the transducer is proposed.

The development of digital measuring systems and signal processing allows the use of many testing frequencies. Adding more frequencies increases the sensitivity and resolution of the ECT systems [10].

In [11,12], a new multifrequency method (MFES-ECT) was proposed. In this system, measurements using many testing frequencies (more than 15) and spectrograms were utilized. This method creates a new opportunity to characterize the defects occurring in the tested materials accurately.

Another approach is to use a square wave pulse instead of a single/multifrequency signal. In this method, pulsed eddy current testing (PECT), the exciting coil is fed by a current pulse. The time-domain response of the probe is measured and further processed. An example of computer simulations of this method is given in [13] and its application to the detection and characterization of hidden cracks around fastener sites is presented in [1]. Additionally, PECT using pulses reduces energy use and thus can be used in portable measuring systems.

Instead of induction coils, a motion against the permanent magnets can be used to induce eddy currents. Moving permanent magnets makes it possible to obtain a high amplitude of eddy currents deep in the material. An example is the system shown in [14] and used to inspect ferromagnetic objects, such as pipelines and heat exchanger tubes, utilizing eddy currents induced by rotating permanent magnets. It contains permanent magnets mounted on the arms, rotating around a shaft placed inside a ferromagnetic pipe. Another transducer, shown in [15], is used to inspect planar conducting plates. It consists of a rotating head with permanent magnets used to generate variable magnetic fields and thus induce eddy currents in the tested material.

This paper presents a novel method of nondestructive testing—the pulsed multifrequency excitation and spectrogram eddy current testing (PMFES-ECT) extension of the multifrequency excitation and spectrogram eddy current testing. The new method uses excitation in the form of pulses repeated at a specified time. The pulses consist of several periods of a waveform containing the sum of sinusoids with a selected frequency, amplitude and phase. This solution allows the maintenance of the advantages of multifrequency excitation and at the same time generates high energy pulses similar to those used in pulse eddy current testing (PECT) and creates good conditions for the accurate detection of flaws located deeply under the surface of the conductive materials.

The concepts and working principles of PMFES-ECT are provided in Section 2. Section 3 shows the main components of the system for testing and validating the PMFES-ECT method, including the description of the sensor and the tested specimen. Based on this, computer simulations were carried out using COMSOL software (the methodology and the results are presented in Section 4), and experimental tests of the system were carried out, the results of which are presented in Section 5. The conclusions are provided in Section 6.

## 2. Pulsed Multifrequency Excitation and Spectrogram Eddy Current Testing

### 2.1. Pulsed Multifrequency Excitation and Spectrogram ECT Method

In the conventional technique, the alternating magnetic field generated by the coil/set of coils acts on the tested sample. Under the influence of this excitation, a secondary magnetic field is created from the eddy currents, which can be measured with appropriate sensors. There are two distinct methods of shaping the current in the excitation coil: the pulse method and the multifrequency method (including the single frequency method). Each has its advantages; i.e., for the latter, a broad spectrum of frequencies makes it suitable for detecting hidden defects, and for the former, a much higher power output and power efficiency.

The pulsed multifrequency excitation and spectrogram eddy current testing (PMFES-ECT) is a novel extension of the MFES-ECT. This method combines the advantages of MFES-ECT and PECT.

**Figure 1:**
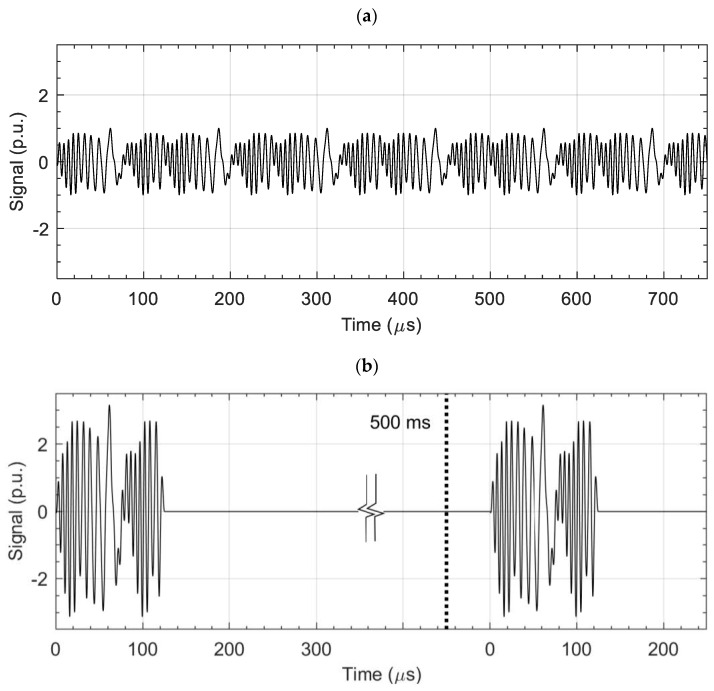
Comparison of excitation voltage for MFES-ECT (**a**) and PMFES-ECT (**b**). There is an interval of about 500 ms between successive pulses in the PMFES-ECT method. The pulse in PMFES-ECT has a power 10 dB more than in the MFES-ECT.

This method uses excitation in the form of pulses. Each pulse consists of several periods of a waveform. The waveform is generated as a sum of sinusoids with selected frequencies, amplitudes and phases. The frequency spectrum of the pulse is identical to the continuous MFES-ECT. Also, a short pulse duration enables a large excitation current, thus detecting deep-lying defects without worrying about overheating the excitation coil. Figure 1 shows the comparison of the excitation signal for the MFES-ECT or PMFES-ECT method. The algorithm for generating the multifrequency pulse is described in the following subsection.

**Figure 2:**
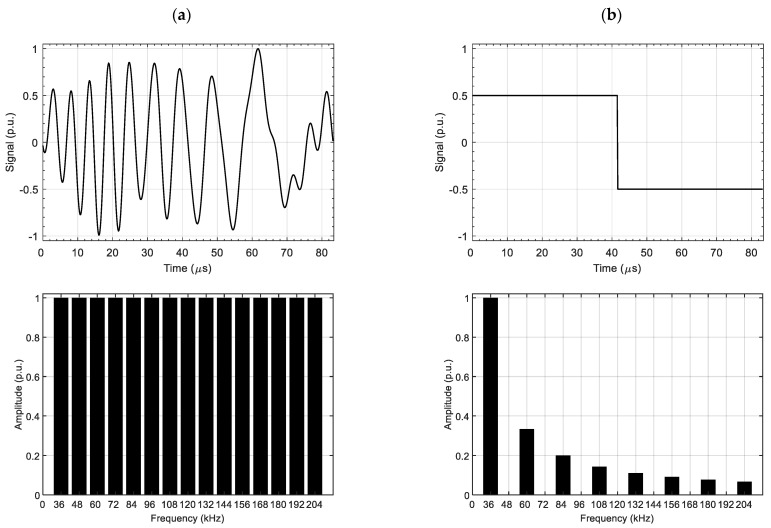
Comparison of the frequency spectrum for PMFES/MFES-ECT (**a**) and PECT (**b**). The signal is at the top and its spectrum (FFT) at the bottom. Signal parameters were selected to obtain the equal amplitude of the first harmonic for both signals.

The spectrum of multifrequency pulse in the frequency range of interest is much richer than in the case of a rectangular pulse. For comparison, in Figure 2, the spectra for the PMFES/MFES-ECT and the PECT waveforms are presented. The rectangular pulse parameters (i.e., the duration and the amplitude of the pulse) used in PECT were selected so that the value of the fundamental (first) frequency amplitude in the spectrum was identical for each type of method.

In the case of the PECT method, successive frequency components have successively lower values. The MFES-ECT/PMFES-ECT methods allow a constant amplitude value in the entire frequency spectrum to be obtained.

### 2.2. Generation of the Multi-Frequency Signal

An exciting multifrequency signal is generated by combining multiple sinusoidal waveforms. The general formula of this signal is following:

(1) $$u_{exct}\left(t\right)=\overset{n}{\underset{i=1}{\sum }}a_{i}U_{i}\sin\left(2\;\pi f_{i}+\phi_{i}\right)$$

where $U_{i}$ is the amplitude of the *i*-th sinusoid, $a_{i}$ is normalization factor $f_{i}$ is frequency and $\phi_{i}$ is the phase angle of *i*-th sinusoid. The factor $\phi_{i}$ is calculated using Equation (2):

(2) $$\phi_{i}=\pi \frac{i^{2}}{N}$$

where *N* is the total number of sinusoids. Setting the parameter $\phi_{i}$ reduces the crest factor of the signal, and thus improves the power delivery to excitations coils. The $a_{i}$ factor is calculated using Equation (3) presented in the following section.

A complex excitation signal containing many sinusoids creates an opportunity to detect all kinds of defects. The frequency range has been selected so that it is possible to detect surface and deeply located defects in tested specimens. Their selection also considered the maximum RMS value of the current flowing in the coil (to avoid overheating). More specifically, the number of frequencies was *N* = 15 and the frequencies were in the range $f_{i}=36,\;48,\;\ldots ,\;204\;\mathrm{kHz}$. The signal amplitude at the beginning and end of a pulse rises and falls linearly to shorten the transition state.

Compared to the continuous wave method (MFES-ECT), the pulse method uses a pulse—a packet constituting a fragment of the excitation wave from the MFES-ECT method. The pulse length is selected empirically to eliminate the transient state in the response signal and obtain good frequency resolution in further analysis.

### 2.3. Maintaining a Constant Amplitude over the Entire Frequency Spectrum

In order to ensure a constant amplitude over the entire spectrum, the excitation signal adjustment/normalization procedure is performed. If the value of *U_i_* from Equation (1) is equal for each frequency, the amplitudes of successive frequencies in the measured signal are not constant. This is due to the variable coils impedance for different frequencies, different signal attenuation, the inductance of connection cables and parasitic capacities.

**Figure 3:**
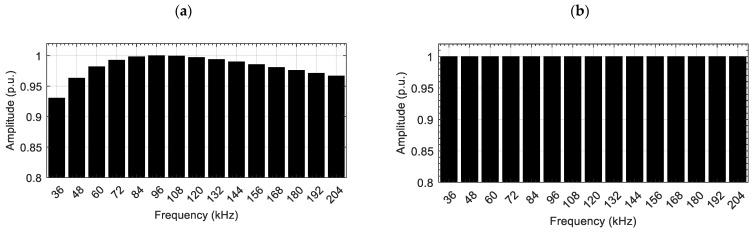
Spectrum comparison for non-normalized (**a**) and normalized (**b**) measured signals.

To mitigate this, we first measured the response to the excitation signal with a constant and equal *U_i_* value in a homogeneous region (without any defects). Figure 3a shows the harmonic amplitudes in the simulated measured signal with no normalization.

Then, we calculated the correction coefficients a_i_ for successive U_i_ values according to the formula:

(3) $$a_{i}=\frac{U_{ref}}{U_{i\;measured}}$$

where *U_ref_* is the reference value we want to acquire, *U_i_* measured is the current value of the amplitude of successive components. The *U_ref_* value is selected so as not to exceed the maximum sensor operating current.

The normalization factor is then used to generate a new excitation signal according to Equation (1). Figure 3b shows the spectrum of the measured signal after normalization in a homogenous area.

### 2.4. Information Extraction and Spectrogram Creation

The signal obtained from the pickup coil, after processing and analysis, is presented in the form of the spectrogram. The spectrogram is a plot of the amplitude of subsequent frequency components of a signal from the coil versus the sensor position. The presented amplitude for each frequency on the spectrogram ($\Delta U_{\mathrm{RMS}}$) is calculated as the difference between the actual amplitude measured at the measurement point and the amplitude measured at the uniform material location.

Selected parameters of spectrogram are defined as:

1. $S_{MAX}$—the maximum value of the spectrogram,
2. $f_{MAX}$—the frequency for which the spectrogram achieves the maximal value
3. $S{\left(f\right)}_{x=X_{MAX}}$—the frequency characteristic at the point $x=X_{MAX}$.

**Figure 4:**
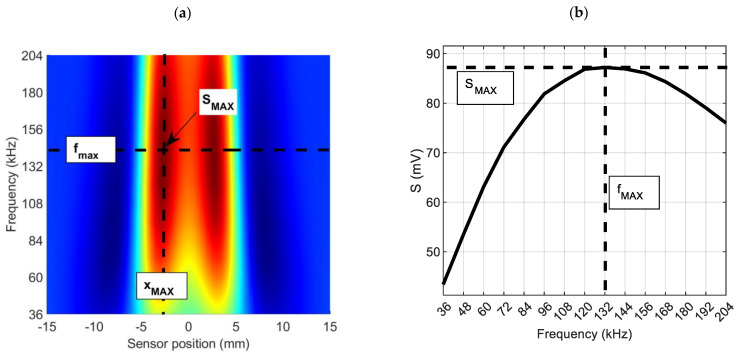
Spectrogram: the two-dimensional plot (**a**) and frequency characteristic for *X_MAX_* point (**b**) with marked parameters (*S_MAX_, f_MAX_*) corresponding to the material defect.

Figure 4 shows the sample spectrogram (in the two-dimensional view) with the above parameters marked. These parameters are utilized in the evaluation of the characteristics of the defect (i.e., position, depth). The estimation of these parameters based on the spectrogram is presented in [11,12].

The measurement procedure assumes that the RMS value of the signal is constant for a given measurement (one pulse). The pulse generator is synchronized with the A/D, the sampling frequency and the number of samples are selected so that a rectangular window can be used in the FFT analysis and there is no spectral leakage.

The signal for each measurement point was decomposed using the FFT algorithm and the amplitudes of successive frequencies were determined. In order to enhance the sensitivity and reduce noises or trends, additional signal processing algorithms can be applied.

**Figure 5:**
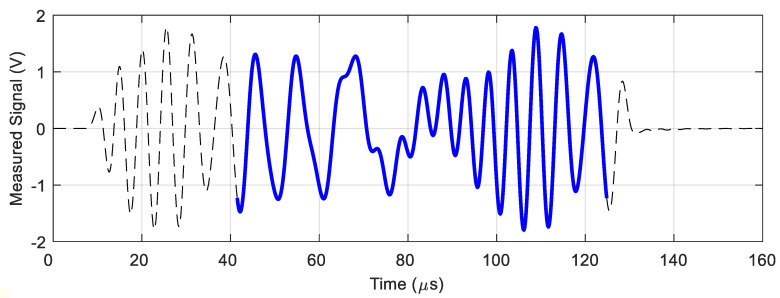
The measured signal on the pickup coil. The blue line marks the signal segment that is subject to further analysis.

Due to the pulse characteristic of the operation, the signal waveforms from the pickup coil contain a transient state. Only a fragment immediately after the end of the transient state was selected for further analysis (determination of the amplitudes of subsequent frequency components). Figure 5 shows an example of the recorded signal waveform and the indication of the part selected for further analysis.

**Figure 6:**
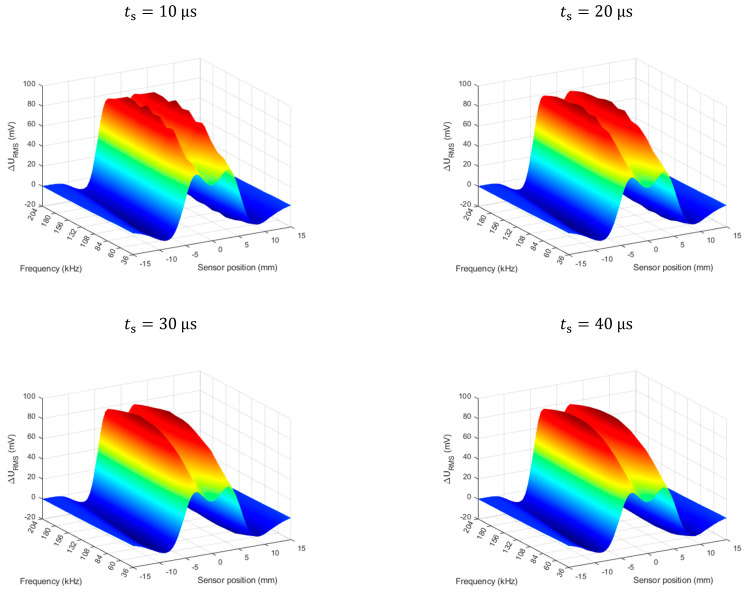
Effect of the transition state on the spectrogram. Flaw depth 60%. The $t_{s}$ is the start time of the data analysis window (the length of the window is constant).

As previously mentioned, an important aspect is to eliminate the transient state when carrying out further analysis. The influence of this state on the generated spectrograms is presented in Figure 6 below.

## 3. Experimental Setup

**Figure 7:**
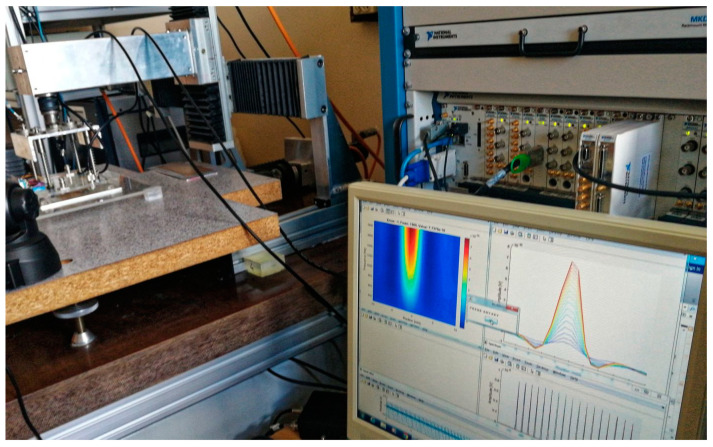
Photo of the experimental setup.

The essential components of the PMFES-ECT measurement system are an excitation signal generator (NI PXI 5422 manufactured by NI based in Austin, TX, USA, sample rate 200 MS/s, 80 MHz bandwidth, 16-Bit waveform generator), a power amplifier (HSA 4101, manufactured by NF Corporation based in Yokohama, Japan, frequency range DC to 10 MHz, slew rate 5000 V/μs, max., current 1.4 A, amplification gain 1–20) used as the excitation coils driver, an eddy current transducer, an A/D capture device (NI PXI 5922 manufactured by NI, Austin, TX, USA, A/D converter maximum sampling rate 15 MS/s, maximum resolution 24 bits) and a PC with dedicated software. A photo of the experimental setup is presented in Figure 7.

The waveform generator outputs pulsed signals to the power amplifier, which supplies the excitation coils in the transducer, thus inducing eddy currents in a tested specimen. The pickup coil measures the effective magnetic field, consisting of a field due to excitation and one caused by eddy currents in the inspected material. The signal from the pickup coil is captured by an A/D converter and saved on a PC for further analysis. Then, the transducer is moved to the next measuring point by the XY linear positioning unit by a step of 0.5 mm. The software on PC manages the whole system. The software also performs information extraction and spectrogram creation on a PC.

A plate specimen with a thickness of 1.25 mm made of INCONEL600 alloy (Nippon Steel Corporation, Tokyo, Japan) with the electrical conductivity *σ* = 1 MS/m is used in this experiment. The plate is 165 mm long, 85 mm wide and contains notches of different depths. The length of the notches is 5 mm and the width is 0.25 mm. The relative depth of the notches varies from 10% to 100% (through the entire thickness of the plate).

**Figure 8:**
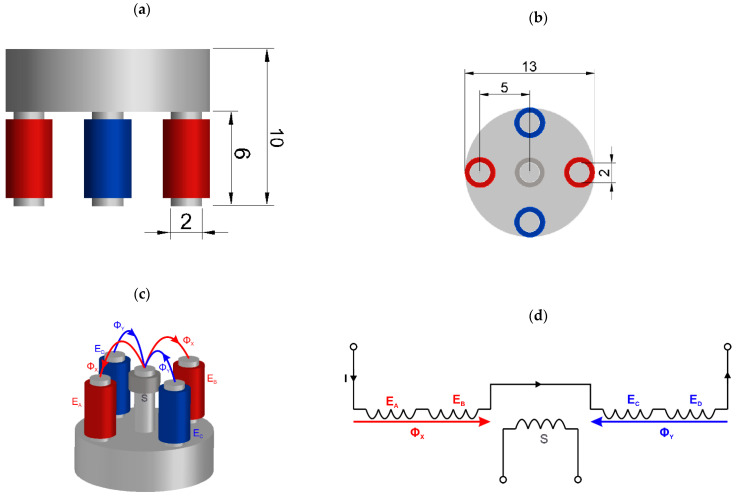
Views of the five columns of ECT transducer used in the experiments: (**a**) front view with dimensions, (**b**) bottom view with dimensions, (**c**) view with magnetic fluxes, the two groups of the excitation coils are marked with red and blue color, (**d**) the simplified electrical scheme of the probe.

Pulsed multifrequency eddy current testing (PMFES-ECT) using an ECT transducer [11], presented in Figure 8, is employed to gather responses from flaws during the movement of the probe. For comparison, the standard MFES-ECT (continuous wave excitation) is also utilized.

The pickup coil (S) mounted on a center column of the 5-column ferrite core measures a differential flux generated by two oppositely oriented pairs of the excitation coils (E_A_, E_B_ and E_C,_ E_D_). Flux produced in the pickup coil by one pair of the excitation coils flows in the opposite direction to the one caused by another pair. The resulting flux in the pickup coil in the equilibrium state is about zero. In the event of a defect in the tested specimen, a signal appears on the measuring coil S.

The ferrite core has a relative permeability of $\mu_{r}=1000$. The exciting coils are driven by a multifrequency signal generated from the arbitrary wave generator and amplified by a high-frequency power amplifier. Dimensions of the sensor are presented in Figure 8a,b. Detailed transducer characteristic is presented in Table 1.

The instantaneous waveforms of induced voltage on the pickup coil are acquired by an A/D converter and saved for further analysis.

**Figure 9:**
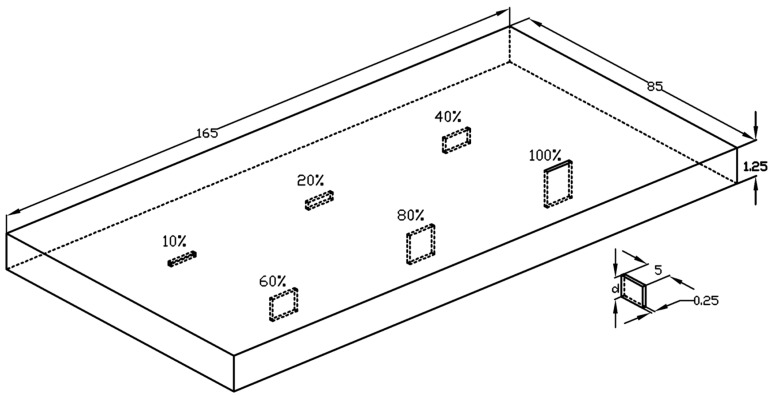
A simplified view of the specimen with artificial flaws.

As mentioned earlier, the plate made of INCONEL600 contains six notches manufactured by the electric-discharge method. The plate is inspected from the opposite side, i.e., opposite of the notches opening (except the 100% notch). The notches have the same length of 5 mm and width of 0.25 mm, while they differ in relative depths d = 10%, 20%, 40%, 60%, 80%, 100%. The view of the specimen is shown in Figure 9.

**Figure 10:**
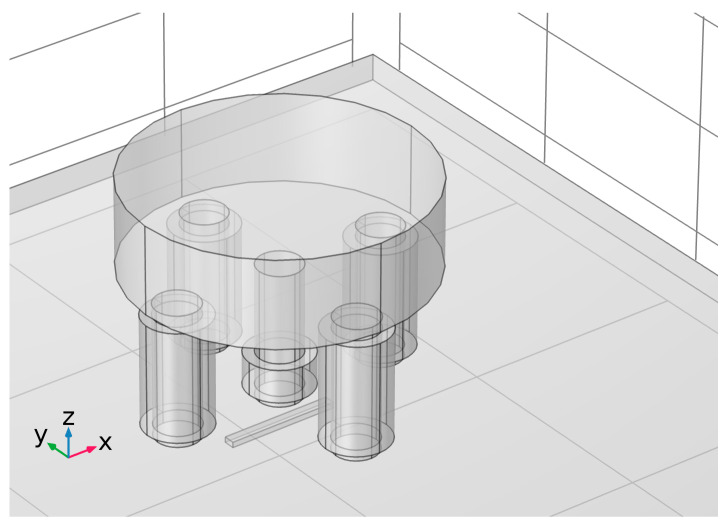
The transducer above the tested specimen with a flaw.

The transducer is placed over the tested specimen (INCONEL600 plate), touching its surface (as shown in Figure 10). Measurements are taken along the defect ranging from −15 mm to 15 mm from the center of each notch using the XY linear positioning unit in 0.5 mm increments.

## 4. Simulation Analysis

### 4.1. FEM Model

**Figure 11:**
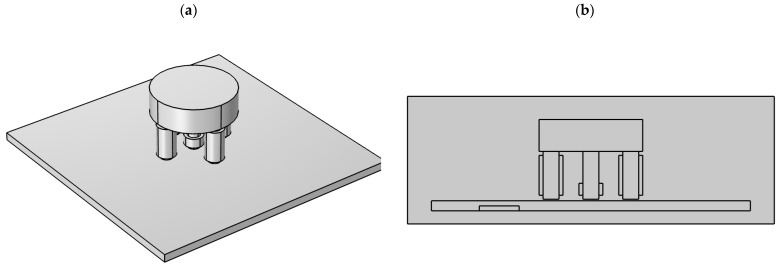
The geometry of the sensor and the specimen with flaw arrangements (the three-dimensional (**a**) and two-dimensional (**b**) configurations).

In order to initially verify the method, FEM two-dimensional and three-dimensional models were made and simulations were carried out in the COMSOL Multiphysics environment. The “magnetic fields” model was used. The size of the mesh elements was selected so as to take into account the skin effect for the highest utilized frequency. Two types of simulations were carried out: “frequency domain” and “time dependent”. Figure 11 shows the model geometry used to analyze the influence of the defect on the distribution of the electromagnetic field and, ultimately, the value of the voltage induced on the pickup coil.

In the simulation experiment, the specimen with dimensions analogous to those in the experimental setup was used, i.e., 1.25 mm thick. The plate conductivity is 1 MS/m, and the relative permeability is 1. The conductivity of the ferrite core is σ=1 S/m, while the relative magnetic permeability is $\mu_{r}=1000$. The conductivity of the surrounding air is 0 S/m and the relative permeability is 1.

The defect dimensions are the same as those presented in Section 3, as are the core dimensions. The flaw length is 5 mm and the relative depth is in the range of 10–100%.

A series of simulations with a different location of the flaw in relation to the sensor was performed—at a distance of −15 mm to 0 mm (the defect was directly under the middle column of the sensor) with a step of 0.5 mm. In order to compare the MFES-ECT and PMFES-ECT methods, a series of simulations, “frequency sweep” and “time simulation”, were performed for each flaw position.

### 4.2. Simulation Parameters and Settings

Frequency domain and time simulations were carried out for each measuring point in COMSOL software. In the frequency domain, simulations were carried out for the frequencies 36–204 kHz, with a step of 12 kHz and a constant amplitude. The simulation results in the form of amplitudes and phases calculated for successive frequencies were saved for further analysis.

**Figure 12:**
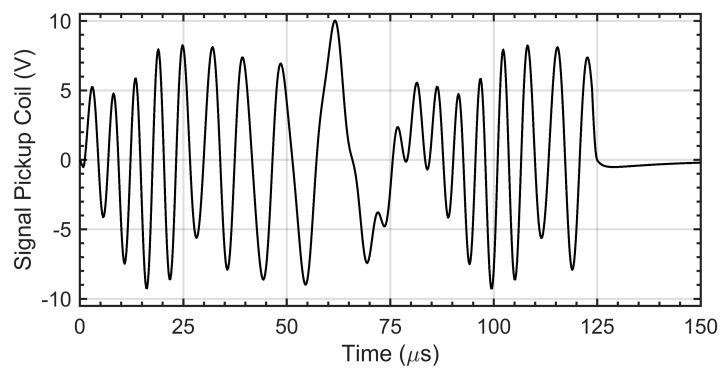
The pickup coil output signal obtained from time dependent simulations. The relative depth of the flaw is 60%.

In the time dependent simulation, the pulse described in Section 2.2 was used as the feeding voltage for excitation coils. The pulse duration was 125 μs, and the entire simulation time was 150 μs, with time step not larger than 0.20 μs. The voltage waveform on the pickup coil was saved for further analysis. An example of the signal achieved from the pickup coil is shown in Figure 12.

### 4.3. Simulation Results

**Figure 13:**
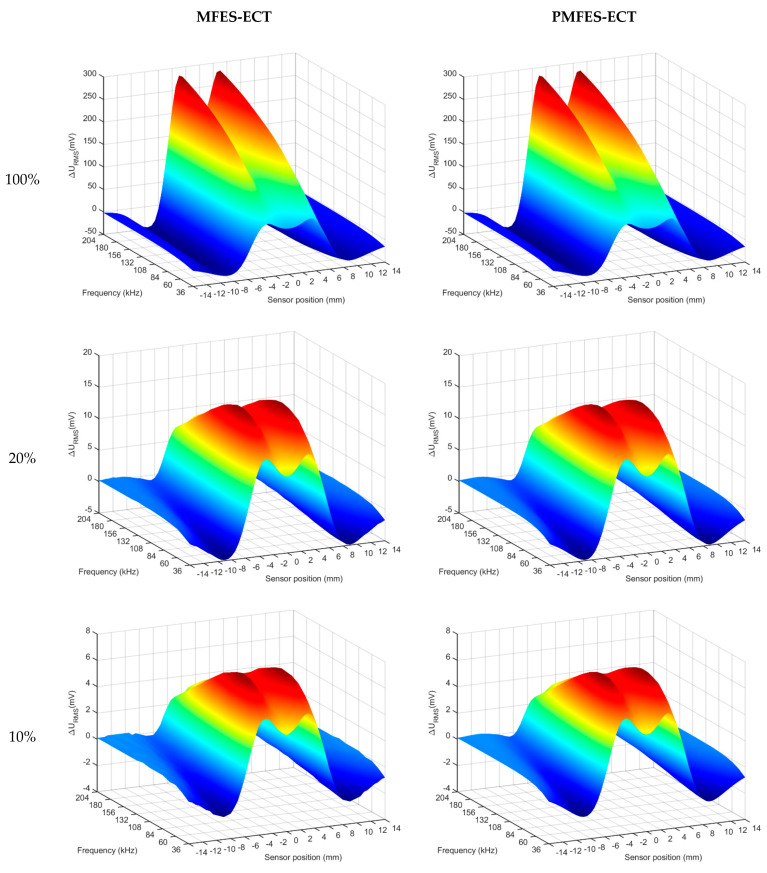
Comparison of spectrograms achieved from two methods MFES-ECT and PMFES-ECT, for various flaws depth.

This section presents simulation results that allow us to compare both methods: PMFES-ECT and MFES-ECT. The simulations were performed for various depths of flaws, according to Section 3. Figure 13 shows the comparison of the spectrograms achieved from the MFES-ECT and PMFES-ECT methods for various flaw depths.

For each defect depth for each method, the $f_{MAX}$, $x_{MAX}$ and $S_{MAX}$ parameters were almost identical. Both methods in the simulation environment provide the same results and enable precise estimation of the length and depth of a defect, and therefore can be used interchangeably.

## 5. Experimental Results

After the validation of the proposed method in computer simulations, physical tests were performed. In the experimental studies, INCONEL alloy samples with notches manufactured by the electric-discharge method were used. The RMS value of the excitation voltage was constant for each depth of the flaw. In the PMFES-ECT method, the RMS voltage value for one period (one pulse) was the same as for the MFES-ECT method.

### 5.1. Experimental Results for Flaws Having Depths in the Range of 10–100%

**Figure 14:**
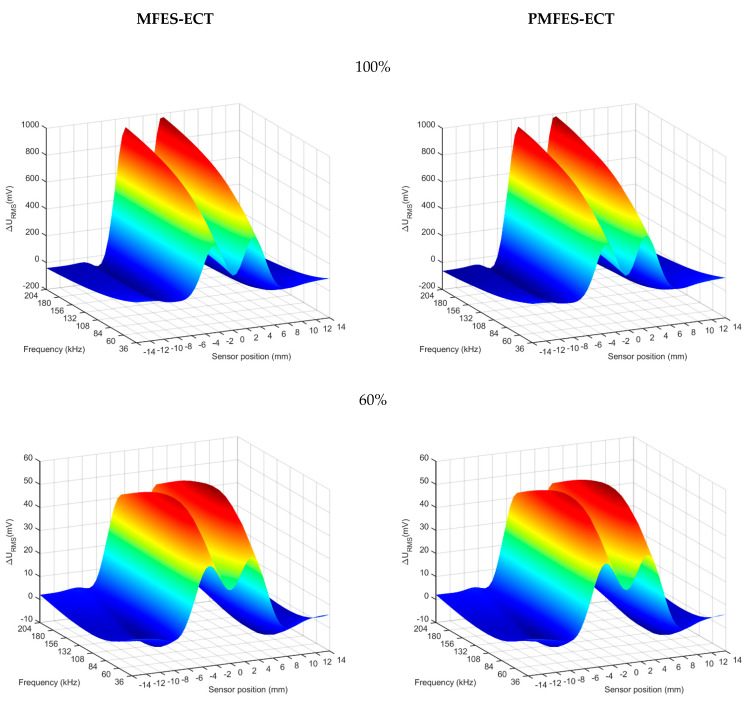

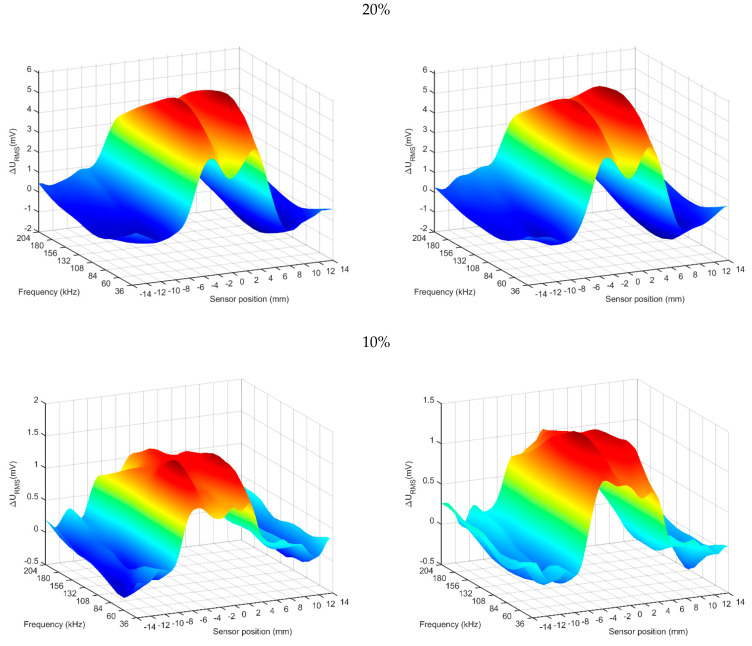
Comparison of the results obtained for the MFES-ECT and PMFES-ECT methods for various flaw depths in the range of 10–100%.

Figure 14 shows a comparison of the results of experiments with both methods. The results are presented for different depths of the flaws.

As in the case of simulation, the spectrogram parameters in the range of 20–100% defect depths are almost identical for the PMFES-ECT and MFES-ECT methods. The detection and characterization of the deepest defect (10%) is subject to high uncertainty due to the distortion of the generated spectrogram. This is due to the high amount of noise in the measured signal. The Table 2 below shows the signal-to-noise ratio (SNR) values for each defect depth.

The pulse method offers the opportunity to improve the SNR value by increasing the pulse power, as shown in the next subsection.

### 5.2. Experimental Results for a Defect with a Depth of 10% at a Higher Pulse Powers

**Figure 15:**
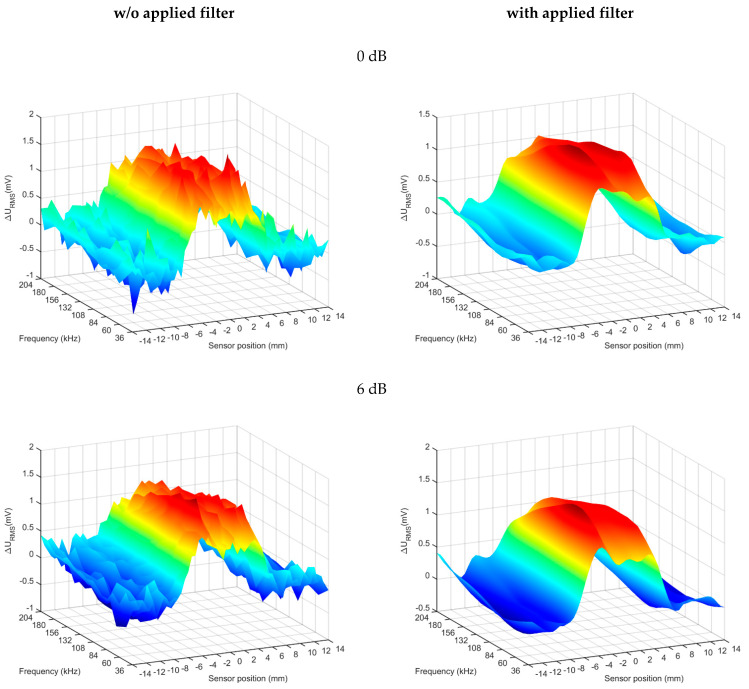

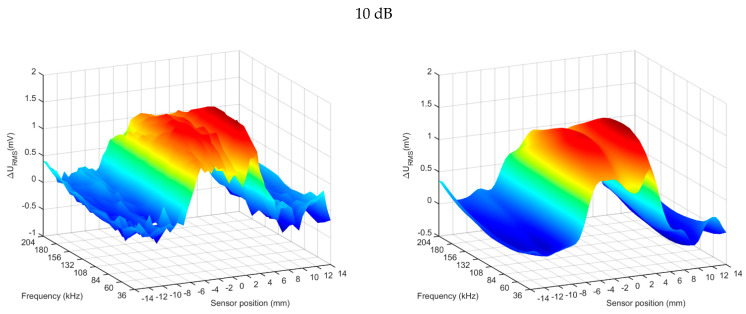
Comparison of the results for the 10% flaw depth for different excitation pulse powers. Results are shown with and without filtering.

In the case of the deepest laying flaw (relative depth of 10%), a series of measurements were performed with a different pulse power value. The 0 dB reference value has been established on the level of previous measurements for the 10–100% defect depth. Additionally, the results were compared without and with the applied filter. The filter type is low-pass Butterworth and was applied to the generated spectrogram in the *x* (position) and *f* (frequency) domains. The results are shown in Figure 15.

The results obtained for a pulse with a reference power (0 dB) show a significant amount of noise. Increasing the pulse power improves the signal-to-noise ratio (SNR) and enables more accurate visualization, defect detection and identification. The use of filters allows you to significantly improve the SNR. The Table 3 below shows a comparison of the SNR values for different pulse powers.

The results of the experiments show that the use of the PMFES-ECT method with increased pulse power and the use of filtering significantly increases the SNR value and the possibility of detecting deeply lying defects.

In the case of a 10% defect, the gain for PMFES-ECT was as high as 5 dB compared to the MFES-ECT method.

## 6. Conclusions

This article presents the novel pulsed multi-frequency eddy current method for nondestructive testing (PMFES-ECT). The method was initially verified by the computer simulations and then experimentally validated.

The proposed measurement method can be utilized for the inspection of conducting materials with different thicknesses. It combines the advantages of the multifrequency and pulse ECT methods. A rich frequency spectrum enables accurate and detailed identification of flaws, and the pulsed operating principle increases the range and detection of deeply located flaws. Moreover, it also reduces energy consumption, which is essential in portable systems.

The spectrogram and peak frequency enable the determination of the depth of the defect and location in examined material, while the maximum amplitude of the spectrogram is correlated with the defect size.

In future work, this method could be used together with artificial intelligence methods to create an autonomous, portable system for detecting and identifying defects.

## Figures and Tables

**Table 1:** Transducer parameters.

Parameter	Value
Coil E_A_, E_B_, E_C_, E_D_ winding turns	25 turns, Φ 0.14 mm
Coil S winding turns	100 turns, Φ 0.02 mm
Core relative permeability	$\mu_{R}=1000$
Maximum working flux density	200 mT

**Table 2:** SNR (dB) values for different defect depths.

Depth	MFES-ECT	PMFES-ECT
100%	24.94	24.86
60%	26.53	26.55
20%	22.87	21.64
10%	14.08	17.55

**Table 3:** SNR (dB) values for different pulse powers with and without filtering for defect depth 10%.

Pulse Power	Without Filtering	With Filtering
0 dB	14.48	17.55
6 dB	16.16	16.50
8 dB	13.68	17.68
10 dB	13.97	19.29

## Data Availability

The data presented in this study are available on request from the corresponding author. The data are not publicly available due to a complicated structure that requires additional explanations.
